# Motivational characteristics of recreational drug use among emerging adults in social settings: an integrative literature review

**DOI:** 10.3389/fpubh.2023.1235387

**Published:** 2023-10-31

**Authors:** Alicja Lojszczyk, Rhonda Wilson, Jessica Wood, Alison Hutton

**Affiliations:** ^1^School of Nursing and Midwifery, University of Newcastle, Callaghan, NSW, Australia; ^2^School of Nursing, Massey University, Wellington, Manawatu-Wanganui, New Zealand; ^3^School of Nursing and Midwifery, Flinders University, Adelaide, SA, Australia; ^4^School of Nursing, John Hopkins, Baltimore, MD, United States

**Keywords:** recreational drugs, illicit drugs, young adult, emerging adult, motivation, decision making

## Abstract

**Introduction:**

Recreational drug use by emerging adults has been identified as an increasingly normalized trend in social contexts. It has been documented that the consumption of these substances regularly occurs at music festivals, raves, nightlife and party settings. While it is known that emerging adults participate in these risk-taking behaviors, what is not known is their motivational characteristics for use. The aim of this review to identify and review literature describing the motivations for recreational drug use and drug choice (excluding alcohol, cannabis and tobacco) by emerging adults in social settings to inform selection of appropriately aligned harm reduction education and health messaging interventions.

**Methods:**

Whittemore and Knafl’s (2005) integrative approach was used to conduct the review. This integrative review was based on a three-step search strategy identifying 2,772 articles published between 2000 and 2022. Eleven studies were included in the review. This review explores the following areas: drug use settings, concurrent drug use, consumer drug knowledge, motives of use including likes and dislikes and peer influence.

**Results:**

A range of factors influence motivations of emerging adults to participate in recreational drug use. Similar to the consumption of alcohol, the use of recreational drugs by emerging adults is motivated by their perceived benefits and personal motivations to achieve euphoria, emotional intimacy, social benefits, peer influence, increased confidence and to decrease inhibitions. The review findings suggest that motivational factors that reinforce recreational drug use correlate with the desire to break away from the mundane by seeking pleasure and for the opportunity to create novel experiences. Beliefs about the positive and negative impacts of drug use, together with the desire to achieve emotional satisfaction influence drug taking activity.

**Conclusion:**

Recreational drug use has become an increased societal norm amongst drug using peer groups and cannot be entirely prevented. It is to be noted that emerging adults have a basic understanding concerning recreational drugs, however, consumer drug knowledge and interventions that target illicit substances is lacking and should be addressed in future research. Festivals, raves and nightlife settings provide opportunity to implement health promotion as it reaches large number of vulnerable individuals in a short period of time.

## Introduction

In comparison to the general population in Australia and internationally, recreational drug use by emerging adults has been identified as an increasingly normalized trend in social contexts (1, 2). It has been documented that the consumption of these substances regularly occurs at music festivals, raves, nightlife (clubs, pubs and concerts) and party settings (3). In 2021 it was estimated 296 million people, constituting 5.8% of the global population engaged in the consumption of illicit drugs (4). Within the European Union, 29% of adults between the ages of 15–64 years acknowledged having used illicit drugs in their lifetime (2). Findings from the 2019 National Drug Strategy Household Survey (NDSHS) identified an increase in illicit drugs use in Australia where 43% (9 million) of the Australian population, reported having used illicit drugs in their lifetime, and 16.4% (3.4 million) reporting use within the last month (5). With the continuous use of recreational drugs and increasing popularity of outdoor music festivals and electronic dance music at nightlife events (including raves and concerts), it is important to identify the motivation for drug use and drug choice among young people (6–8). Hutton et al. (9) states that recreational drug use by emerging adults suggests that participants aged 30 and younger are more likely to engage in risk taking behavior resulting in increased presentation rates to hospitals. This Australian based study found that in comparison to other outdoor events there was a higher rate of drug consumption at outdoor music festivals (9). While it is known that emerging adults participate in these risk-taking behaviors, what is not known is their motivational characteristics for use. The review to identify and review literature describing the motivations for recreational drug use and drug choice by emerging adults at music festivals, nightlife and party settings to inform selection of appropriately aligned harm reduction education and health messaging interventions. Alcohol, tobacco and cannabis were excluded due to these substances being well studied. With the continuous emergence of new psychoactive substances, growing support of drug testing exploration within Australia and recreational drug use being understudied in the research community, this is the manuscript’s area of focus. Motivations in association to recreational drug use is important to evaluate as motivation is deemed as the undeniable driving force behind all behavior (7, 10).

### Background

The term motivation is a psychological process used to define the process that activates, directs, and maintains goal-directed behaviors until a particular goal is achieved (11–13). The term motivation is frequently used to describe ‘why’ a person undertakes a certain activity and it is perceived as the driving force behind human actions involving factors that direct and maintain these goal-directed actions which in this case is recreational drug use by emerging adults (11, 13). Recreational drug use motivation can be sustained by positive reinforcements, expectations and the perceived value of use such as drug intoxication (14, 15). The motivational process involves biological, emotional, social and cognitive forces that activate the behavior and explains why an individual undertakes a certain behavior. These motives however can not be observed, rather, conclusions can be made based on the behaviors observed (13). Literature has identified that five distinct or conceptual dimensions to assess motivations to use drugs (16). Motivations explored included: to increase creativity (16), increase social cohesion (17), increase positive affect and reduce negative affect (18).

For the purpose of this review, motivational use of ‘recreational drugs’ are defined as legal and illegal substances that are taken for enjoyment, pleasure, or leisure purposes without medical reasoning to induce a heightened or altered state of consciousness (19). In these settings, party drugs traditionally refer to stimulant and hallucinogenic drugs commonly used to enhance the user’s experience (7). Excluding alcohol and tobacco, current literature recognizes that cannabis, 3,4-methylenedioxy-methamphetamine (MDMA/ecstasy), methamphetamines, amphetamines (speed), cocaine, lysergic acid diethylamide (LSD), psilocybin (magic mushrooms) and ketamine are the most used recreational drugs by emerging adults (18–30) in Western countries including Australia and Europe (2, 7, 20–22). Social settings where emerging adults commonly consume recreational drugs include music festivals, dance venues, dance parties/raves, nightlife, home and party settings (1, 2, 21, 22). Rave subculture originated from the late at 1980s acid house parties that are all-night dance parties commonly held in covert locations that consists of live deejay-mixed techno and electronic dance music (EDM) (23, 24). Recreational drug use within music festival environments is reportedly higher than those seen outside of these environments (20, 25–27).

Prior to the global COVID-19 pandemic, recreational venues provided a place of entertainment for emerging adults to socialize and come together to appreciate and enjoy music (28). Within nightlife and festival environments, attendees may engage in risk taking behaviors, including recreational drug use. Drug consumption patterns can differ amongst venues and their participants as drug preference and use can be associated with music genres and sub-cultures as explored in Lim et al.’s Australian study (20). For example, dance/house music genres are associated with ‘rave drugs’ including MDMA, amphetamines, LSD, cocaine and ketamine whereas pop music attendees consumed lower amounts of recreational drugs (20).

Recent studies recognize that festival and rave attendees who used drugs in these environments had also reported using these substances earlier in life (9, 29–31). Normative behaviors for these participants often consists of consumption of recreational drugs in familiar environments including nightlife and party settings. This finding acknowledges that music venues do not promote illicit drug use as attendees bring their common day to day norms of recreational use into these settings (9, 29, 30). Nightlife venues including nightclubs, pubs, outdoor music festivals and raves provide a diverse range of music preferences with informal access to legal and illegal substances commonly reported by emerging adults (32).

The behaviors of emerging adults including recreational drug use, are associated with societal norms and normative behaviors in social settings (28). Internationally, recreational drug use has been acknowledged to be associated with various public heath-related issues (28). Investigating these settings and motivations for recreational drug use can inform health messaging and harm minimization interventions in the context in which emerging adults use drugs and drugs of choice.

### Aim

The aim of this review was to critically evaluate and synthesize this area of literature describing the motivational characteristics for recreational drug use and drug choice (excluding alcohol, cannabis and tobacco) by emerging adults (aged 18–30 years) at music festivals, nightlife and party settings to identify the gaps in research and inform selection of appropriately aligned harm reduction education and health messaging interventions.

## Methods

An integrative review method was chosen for this study as this process facilitates the inclusion of studies of varied methodological approaches to develop a comprehensive understanding of the motivations of recreational drug use and drug choice by young and emerging adults (33, 34). The review process was conducted in five stages: (i) problem identification; (ii) systematic search of literature; (iii) data retrieval; (iv) article evaluation; and (v) data analysis and presentation (33). This method accommodates the inclusion of a broad range of literature including both experimental and nonexperimental studies suitable for addressing the research aim to inform appropriate harm reduction education and health messaging interventions. Integrative reviews are grounded on an evidence-based-practice approach which provides structure and rigor to the data analysis stage of the review (33). The method of the preferred reporting items for systematic reviews and meta-analysis (PRISMA) statement (35) checklist was utilized when undertaking the research process.

### Problem identification

Emerging adults are people aged between 18 and 30 who are in the transitional period of emerging adulthood (3, 36–38). During this phase, personal and peer-group identity is formed by the adaptation of developmental, social, health and environmental influences. These influences can be linked to the engagement of risk-taking behaviors such as recreational drug use (3, 39, 40). Therefore, this research aimed to evaluate and synthesize the motivational characteristics for recreational drug use and drug choice by emerging adults.

### Literature search

The aim of this review was to uncover and examine primary research that describes the motivations for recreational drug use and drug choice by emerging adults at music festivals, nightlife and party settings. Studies published from 2000 to June 2022 were identified using a comprehensive in-depth search of databases. In Westernized societies, the accessibly and prevalence of illicit drugs at music festivals and raves has increased over the last 20-years, so this time frame ensured that relevant studies were identified. Relevant keywords, index and MeSH terms were applied to identify appropriate search terms as seen in Table 1. Databases searched include CINAHL complete, EMBASE, Medline, EMcare, the Joanna Briggs Institute (JBI EBP), SCOPUS, Sociology Source Ultimate, Academic Search Ultimate, and PsycInfo. Reference lists of all identified reports and articles were then searched for additional studies.

**Table 1:** Search terms.

(motivate* or “decision making” or decision* or prompt* or drive* or intention or intent* or inspir* or influence* or lead* or persuade* or predetermine* or sway* or trigger* or risk tak* or health knowledge, attitudes, practice)
AND
(“recreational drug*"or “designer drug*” or “party drug*” or “illicit drug*” or “illegal drug*” or “illicit substance*” or “illegal substance*” or “club drug” or “synthetic drug*” or “psychoactive drug*” or heroin or MDMA or ecstasy or molly or marijuana or cannabis or THC or cocaine or speed or amphetamine* or tranquillizers or hallucinogens or LSD or acid or mushroom* or ketamine or GHB)
AND
(“young adult*” or “emerging adult*” or “young people” or adolescen* or “18–30 years” or “college student”) drug users (psychology)
AND
(“music festival*” or rave* or “mass gathering*” or “electronic dance music” or EMD or festival* or “or dance part* or nightlife or nightclub* or bar or bars or pub* or club* or disco* or hotspot*)

**Figure 1:**
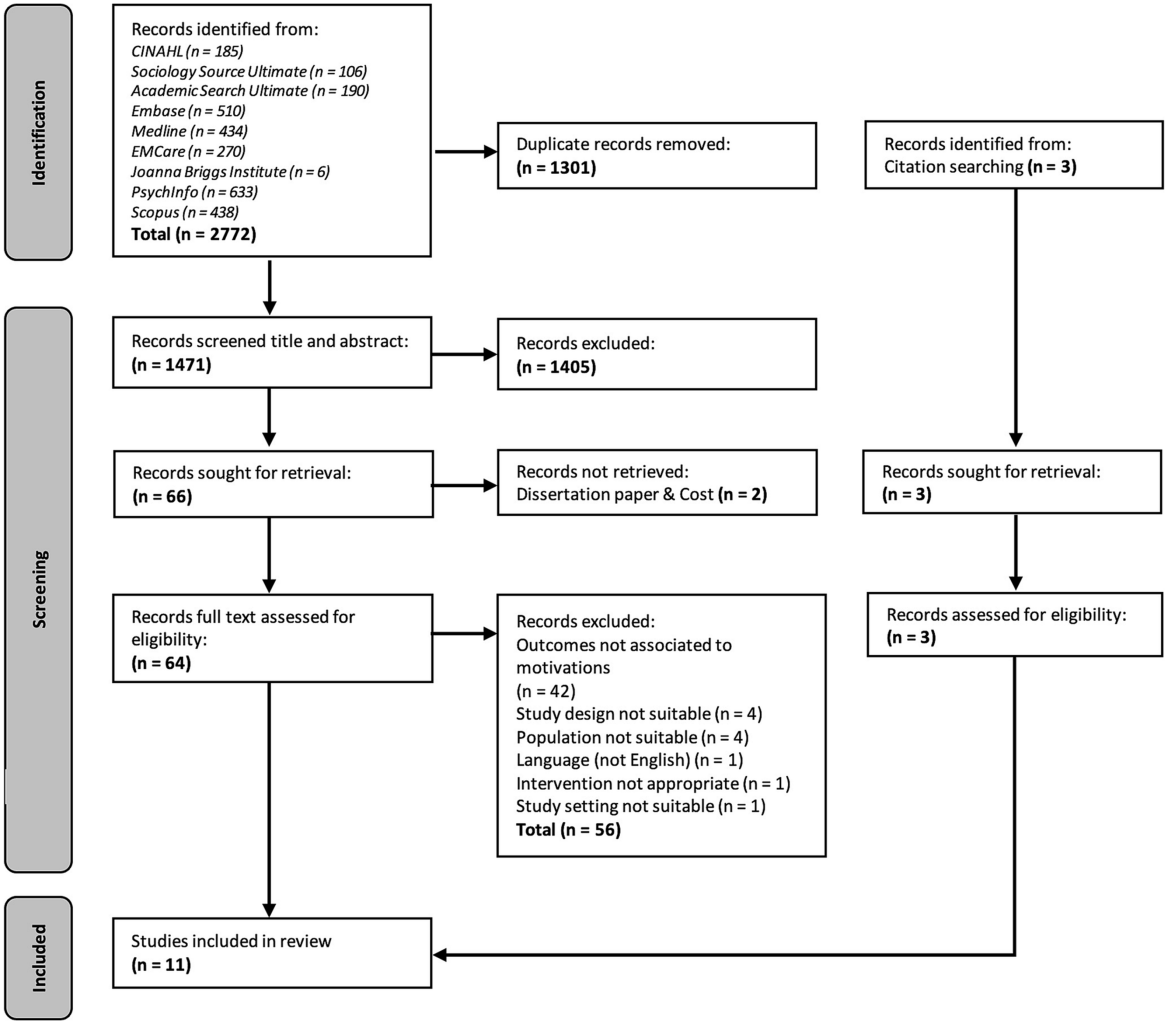
Preferred Reporting Items for Systematic Reviews and Meta-Analyses (PRISMA) chart (35).

The keyword search in electronic databases identified 2,772 articles. Most articles were excluded during the initial review, either due to duplication (*n* = 1,301) or being deemed irrelevant following a screening of study titles and abstracts against the inclusion and exclusion criteria (*n* = 1,407) as displayed in Figure 1. All identified articles selected for inclusion were in English, presented original research, published from between 2000 and 2021 (current) and included the following:

1. Participants: Individuals (aged 18–30) who participate in recreational drug use in nightlife, rave, party and social settings were included.
2. Design: This review included primary studies and grey literature that evaluated decision-making, intentions and motivations in association to illicit drug use.

Publications selected for retrieval were assessed against the inclusion and exclusion criteria to ensure relevant studies were included in the review. If the title or abstract eligibility was unclear, any queries were discussed with experienced study co-authors to ensure quality control. Studies were excluded if:

1. Participants were not within the targeted age group
2. The study focused on drug dependence or treatment for illicit drug use
3. Drugs used for medicinal purposes (prescribed and treatment medications)
4. The study involved athletes using illicit substances for enhancement purposes
5. The study only focused on alcohol, tobacco or cannabis use.

Figure 1 shows the PRISMA (Preferred Reporting Items for Systematic Reviews and Meta-Analyses) flow diagram of the integrative review method (35). As a result of this initial review process, 67 articles were deemed relevant and included in the study. Upon evaluation of the full text, a further 56 studies were excluded when evaluated against the study’s inclusion and exclusion criteria. This resulted in a total of 11 articles remaining for critical appraisal, analysis and discussion. All articles were double reviewed (AL, AH) for quality assurance.

### Critical appraisal

Out of the 11 articles included in this review, six studies were qualitative (41–46), four were quantitative (47–50) and one study consisted of mixed-method design (51). The critical appraisal skills program (CASP) (52) principles were used to determine validity, methodological rigor and data relevance as seen in Table 2. Whilst all articles discussed were strong in their methodological approach, studies were mostly descriptive. Most studies included in this review acknowledge that sample numbers were limited in size or had a low response rate limiting definitive study conclusions and representable samples (42–46). Small sample sizes are common for qualitative research analysis as this research method focuses on developing an in-depth understanding of a particular phenomenon in contrast to quantitative studies where larger or statistically significant quantity of data is required (53). It is also acknowledged that one article included in this review was identified as a pilot study (47) and one study did not disclose research limitations (41). Although this literature review was primarily seeking information about recreational drug use by participants aged between 18 and 30, it should be noted that only five studies specifically used participants in this age group (42–45, 51). The remaining six studies included participants of mixed ranges from 16 to 40+ years (41, 42, 46–50). These findings reveal that while there are few published research studies available internationally, and the available evidence relies on small data sets, thus it is significant to examine this topic in more depth and further research.

**Table 2:** CASP tool for studies.

Author and year	Clear aims and focus of study	Methodology appropriate	Research design appropriate	Appropriate recruitment strategy	Appropriate data collection	Relevant outcome factors identified	Data results precise and consistent	Clear statement of findings	Ethics considered	Study findings fit with known evidence	Implications for modern practice
Biolcati and Mancini (2018) (47)	Y	Y	Y	Y	Y	Y	Y	Y	Y	Y	Y
Boys et al. (2001) (48)	Y	Y	Y	Y	Y	Y	Y	Y	Y	Y	Y
Duff (2005) (49)	Y	Y	Y	Y	Y	Y	Y	Y	Y	Y	Y
Duff et al. (2007) (41)	Y	Y	Y	Y	Y	Y	Y	Y	Y	Y	Y
Fendrich et al. (2003) (50)	Y	Y	Y	Y	Y	Y	Y	Y	Y	Y	Y
Fox et al. (2018) (42)	Y	Y	Y	Y	Y	Y	Y	Y	Y	Y	Y
Levy et al. (2005) (43)	Y	Y	Y	Y	Y	Y	Y	Y	Y	Y	Y
Parks and Kennedy. (2004) (44)	Y	Y	Y	Y	Y	Y	Y	Y	Y	Y	Y
Peters et al. (2008) (45)	Y	Y	Y	Y	Y	Y	Y	Y	Y	Y	Y
Smirnov et al. (2013) (51)	Y	Y	Y	Y	Y	Y	Y	Y	Y	Y	Y
Ter Bogt and Engels (2005) (46)	Y	Y	Y	Y	Y	Y	Y	Y	Y	Y	Y

### Data analysis

All data from primary sources were sorted, coded, categorized and summarized into the following areas: study setting, study type, sampling method, participant demographics and findings. The primary data was then descriptively coded and organized into the appropriate themes relevant to the review’s aim. To systematically interpret and analyze source findings, the data was organized into the following themes: recreational drug use (11 studies), concurrent drug use (*n* = 4), drug use settings (*n* = 3), knowledge and harm reduction (*n* = 4), desirable (*n* = 9) and undesirable (*n* = 6) reinforces of use, peer and social influence (*n* = 7) as displayed in Table 3.

**Table 3:** Visual comparison of study themes.

Study	Themes							
	Motivational characteristics	Drug use settings	Concurrent drug use	Consumer drug knowledge and harm reduction	Desirable effects from use	Undesirable effects from use	Normalization of use	Peer and social influence
Biolcati and Mancini. (2018) (47)								
Boys et al. (2001) (48)								
Duff. (2005) (49)								
Duff et al. (2007) (41)								
Fendrich et al. (2003) (50)								
Fox et al. (2018) (42)								
Levy et al. (2005) (43)								
Parks and Kennedy (2004) (44)								
Peters et al. (2008) (45)								
Smirnov et al. (2013) (51)								
Ter Bogt and Engels (2005) (46)								

### Participant characteristics and sampling

A total of 2,529 participants were comprised across all 11 studies included in this review. Among the studies, 47.4% (*n* = 1,198) of participants were male, 40.9% (*n* = 1,034) were female and 11.7% (*n* = 297) were unspecified (Supplementary Table S1). In relation to recreational drug use, four of the studies examined the use and prevalence of MDMA (41, 43, 45, 51), six studies explored multiple ‘club drugs’ including amphetamines, LSD, ketamine, cocaine and GHB (42, 44, 47–50) and one study examined concurrent drug use with MDMA (46). A variety of recreational drug use settings were noted across the studies including: the general public (41, 44, 48, 50, 51), rave/dance parties (45–47), music festivals (42), nightclubs (bars and clubs) (49) and colleges (43).

### Findings

The literature review of 11 articles revealed that recreational ‘party’ drugs used included: MDMA (ecstasy), LSD, cocaine, ketamine, amphetamines, psilocybin and GHB (41–51). Other licit and illicit substances used amongst participants included; alcohol, tobacco, marijuana, opiates, barbiturates, prescription medication, inhalants and sleeping pills. The average age of first-time drug use by participants was between the ages of 17–19 (41, 44, 49). For overview of the main findings please see Table 4.

**Table 4:** Overview of main findings.

Motivational characteristics of use	Emerging adults were often driven by curiosity and initial drug use was opportunistic rather than planned. Gender differences in age of first drug use were not significant, whereas motivations and quantity of drug use and desired outcomes differed between males and females. Rave attendees, exhibited greater drug involvement, reporting more frequent and prolonged use.
Concurrent drug use	MDMA was combined with LSD, cannabis, amphetamines, cocaine, psylocibin, and ketamine. Substance quantities were influenced by specific events and desired outcome.
Drug use settings	Drug use patterns were individualized but used in social settings including parties, festivals, and nightlife venues; prevalence of use differed amongst settings. MDMA was consistently popular in multiple locations (Bologna, Melbourne, Colorado, New York, Amsterdam), but British and American studies highlighted LSD, amphetamines, and cocaine as more prevalent.
Consumer drug knowledge and harm reduction	Participants often relied on peer-to-peer education and peer experiences for reliable drug information due to pill testing constraints. Participants supported harm reduction approaches that provided clear, non-judgmental, evidence-based information.
Desirable effects	Recreational drug use was driven by the desire for positive intoxication effects, with different substances consumed for intoxication effects. Motivations to use included enhance experiences, intoxication, euphoria, curiosity, social bonding, escapism. Social-recreational goals played a significant role in motivating drug use and contribute to the normalization of use in social contexts.
Undesirable effects	The illegal status of drugs did not deter consumption but lead participants to be more cautious. Negative reported effects included temperature deregulation, bruxism, nausea, vomiting, headaches, anxiety. After drug use, participants reported “comedown” hangover effects.
Peer and social influence	Peer influence and social environments play a significant role in motivating recreational drug use. Higher levels of peer intoxication correlated with increased use by participants.

#### Motivational characteristics of recreational drug use

Prior to first drug encounters, emerging adults expressed curiosity about drug use for a prolonged period after observing peer’s intoxication experiences (41). First time encounters of recreational drug use were explained to be incidental and opportunistic rather than planned (41).

It was identified that age of first drug use did not differ by gender (44) and there were no gender differences in the number of different drugs ever used except for amphetamines which was more likely to have been used by females (48). The average length of ecstasy use was recognized by studies to be 2.9 years (44, 51). Quantity of use associated with the following variables including gender (46), socio-recreational goals (51), educational level, use amongst friend and party type (46, 51). Motivations for drug use and the desired outcomes were identified to be different amongst males (e.g., improve effects) and females (e.g., stay awake) (48). Amongst all participants in the study, motivation of use was driven by the desired outcome of intoxication was relevant to increased consumption (seeking euphoria and sexiness) or decreased consumption (wanting to be social and flirtatiousness) (46).

In comparison to the rest of the sample population, rave attendees who participated in recreational drug use were more likely to be male and of a younger age group (50). Rave attendance was also a marker of greater motivation to participate in more intense drug involvement, irrespective of whether club drugs were specifically used at the last venue of attendance. Male rave participants often reported motivational use of MDMA more frequently, more pills per occasion and consumption for a longer period.

Differences between genders after use also varied with females generally experiencing more perceived negative consequences including depression, nausea, dizziness and headaches (50). The LGBTQI+ population had a higher prevalence of recreational drug use in comparison to the rest of the study sample (50). Involvement amongst LGBTQI+ female participants was higher than LGBTQI+ males (50).

#### Concurrent drug use

Polysubstance, concurrent, simultaneous or multiple drug use is defined by the World Health Organization (WHO) as “the use of several substances by an individual, often at the same time or sequentially usually with the intention of improving, increasing or counteracting the effect of another drug” (54). This was investigated by five studies included in the review (41, 43, 46, 47, 50). One study only focused on poly-substance users as specified by their inclusion criteria (41). For example, MDMA was noted to have been consumed with other drugs including LSD, cannabis, amphetamines, cocaine, psylocibin and ketamine (46, 47). The quantity of substances consumed was determined by the type of event attended and the desired intoxication effects (43, 46, 50). As seen in the study by Fendrich et al. (50), participants who attended club parties commonly consumed less MDMA than participants who attended a ‘trance’ or ‘hard-core’ rave parties. Thus, concurrent use was not a uniform phenomenon with a variety of substance mixtures, event types and intoxication goals noted.

#### Drug use settings

Club drugs and party drugs are most commonly used in group settings (41, 47). The prevalence of the drugs consumed varied according to the study locations, distribution, the drugs available, and the associated expense of these substances. Five studies identified MDMA as the most popular drug of choice [Bologna (Italy) (47), Melbourne (Australia) (49), Colorado (United States) (42), New York (United States) (44), and Amsterdam (Netherlands) (46)]. However, findings from a British and American study presented different findings and documented a wider prevalence of LSD (50), amphetamines and cocaine (48) amongst participants. In the Australian study (41) ecstasy was identified as a social drug for party settings and the frequency of use increased over the summer holiday period (December–January). Specific and individual patterns of use were reported as preferential for weekends at parties, private gatherings or other ‘specific event’ environments including festivals and nightlife settings. Nightlife and party settings where drugs were consumed included: house parties, bars, nightclubs, raves and discos; prevalence of use also varied amongst these settings (47, 50).

#### Consumer drug knowledge and harm reduction

Drug knowledge in this review was defined as the individual’s understanding of both the positive and negative effects of drug consumption. Five of the 11 studies addressed this concept of consumer knowledge in association to recreational drug use (41–43, 45). The studies identified common useful sources of information for users about drugs and what to expect from them. This included asking friends, and accessing online articles, trusted websites, and web forums (41–43). Participants from only one study (41) made specific reference to ‘peer’ led harm reduction organizations as important sources of information for users. Participants were also interested in accessible information from doctors and research scientists about the associated harms of drug use. Web forums were a valued source of information as they provided individuals with a platform to share their own experiences to ‘learn from each other’ (41, 43). Findings from these studies showed that most recreational drug users had little to no knowledge of the mechanisms and effects of the drugs they were planning to consume, with only a small number of participants rating themselves as experienced or knowledgeable users (42).

Another avenue to increase consumer awareness and knowledge about drugs of consumption is the availability of pill testing. Pill testing was a known harm reduction strategy among participants in some studies, however this was not commonly utilized by participants due to limitations of accessibility and/or availability of testing facilities (42, 43). Limitations included cost and inadequate results from color metric testing, which only assesses the purity of specific substance rather than potential additives (42, 43). As an alternative to using pill testing resources and facilities, emerging adults preferred to rely on the procuring the substance from a trusted source (42) and shared commentary about the experiences of peers who had previously taken a pill from the same batch acting as an endorsement of the substance’s safety which reassured users (41, 42).

Participants who took part in recreational drug use on a semi-regular basis liked to be informed about the drugs they were consuming (41). Harm reduction approaches were strongly supported by participants however discrepancies were reported in association to the quality and accuracy of participants drug and harm reduction knowledge (41, 43). Participants displayed interest and support for harm reduction and prevention messages that provided clear and balanced information to users about the nature of recreational drug use and the related risks and harms (41). Of interest to this population was information on how to use these drugs more safely especially if these approaches were relevant, non-judgemental and evidence-based (41). Harm reduction resources and approaches were not commonly specified amongst participants of the reviewed studies and remains unclear how accessible this information is (41, 42, 51).

Peer to peer education was used by participants to identify common dangers and risks associated to recreational drug use, however it was recognized that shared information was not always accurate (42, 45, 51). Web forums (41, 43) including *smilepolice* (41), *pillreports.com* (45), and peer-to-peer educations programs provided by *Ravesafe*, *Enlighten*, (41) were reported as effective means to acquire trustworthy sources for drug information. Dangers included environmental hazards, drugs containing harmful adulterants and the challenge of achieving appropriate dosing (42). The only government-funded harm reduction campaign mentioned within the studies was the pill testing facilities conducted by the Dutch Trimbos Institute in the Netherlands (45).

#### Desirable effects from recreational drug use

Recreational drug use by emerging adults is strongly driven by the motivation and desire to achieve the positive intoxication effects with certain substances aimed at improving and enhancing their personal experiences (43, 46, 48, 49). Recreational drug substances can be divided into different categories including stimulants (amphetamines, cocaine), empathogens (MDMA), psychedelics (LSD, psilocybin), depressants (alcohol and benzodiazepines), opioids (codeine, oxycodone, heroin) disassociates (ketamine) and cannabinoids drugs (cannabis, medical and synthetic cannabis) (25). In the identified studies, participants were motivated by positive effects such as: achieving drug intoxication and euphoria (41–46, 48, 50), curiosity (41, 43, 45, 47), peer enjoyment and social connection (41–43, 45–48, 50), avoid boredom (42, 43, 47), escape reality (42, 43), improve sociability (46) and enhance pleasurable sexual experiences (43, 46, 48, 50). Most participants across three studies acknowledge that recreational drug use had become a normalized aspect of social outings to clubs and festivals (41, 42, 49). Social-recreational goals, which included improving current relationships were identified as common motives to use recreational substances (42, 44, 51).

#### Undesirable effects from recreational drug use

Although law enforcement presence may play a role in the consumption of illegal substances, little was mentioned within the reviewed studies. The illegal status of drugs did not deter recreational use, however, it made participants more mindful of their drug intake (41). For example, in the Netherlands although recreational drug use is illegal there are no criminal consequences associated to carry drugs for personal consumption (46). Because of this, rave attendees continue to use recreational drugs (46). Participants who frequently used drugs were aware of common undesirable outcomes related to the risks and harms associated with their consumption. During drug intoxication, negative effects were mentioned by participants in the various studies including temperature deregulation and bruxism (teeth grinding) which are common side-effects of stimulant drug use (55). The reviewed literature identified that these effects primarily occurred following the consumption of ecstasy, followed by ketamine and LSD to a lesser degree (42, 44). The studies indicated that participants reported negative symptoms including nausea, vomiting, headaches, stomach pains, anxiety, confusion, panic attacks and paranoia, which were attributed to drug use (41, 42, 44, 49, 51) Participants who consumed cocaine mentioned feelings of apathy as a negative side effect manifesting following the ‘high’ or intoxication period caused by the drug (42). Undesirable outcomes from drug use in the days following drug use consisted of negative/depressive thoughts, irritability and mood swings following consumption of MDMA and LSD (41, 42, 44, 46, 49, 51). The most frequently reported harm following acute intoxication by participants was the sadness hangover or ‘the comedown’ associated with having an impact on commitments and personal relationships with friends, partners, and families (41). The degree of harm and concern ascribed varied considerably amongst participants with one participant describing it as debilitating (41).

#### Peer and social influence

Peer influence and the social environment have been identified as playing a crucial role in sourcing recreational drugs, increased curiosity and in the motivation and initiation of recreational drug use among emerging adults (43, 47, 49, 51). Witnessing friends use illicit drugs and observing them enjoy themselves while intoxicated was identified as a significant factor in the initiation and the continued drug use (46, 47). Multiple studies identified that levels of intoxication and concurrent drug use amongst peers had a positive correlation with one’s current and future drug use (41, 45, 46, 51). The impulse of social pressure and normative conformity to participate in recreational drug use was also disclosed by a small percentage of participants as influencing their decision to use recreational drugs (43, 46). It was also identified that social-recreational goals in association to dance music events played a role in drug quantity and consumption patterns. For example, this can be seen through participants attending electronic/dance music or hardcore events were more likely to participate in intermediate or high use drug trajectories in comparison to club and mellow pub/partygoers (46, 51).

## Discussion

The age of initiation for recreational drug use was between 17 and 19 years was reported by Duff et al. (41), Parks and Kennedy (44) and Duff (49), supported research by the European Drug Report (2) and World Drug Report (4). Given the motivations of use individuals often transition from legal substances (such as tobacco and alcohol) to illicit substances during the period of experimentation during adolescence and emerging adulthood (56–58). Emerging adults are aware of the negative outcomes associated with recreational drug use but continued to participate due to their beliefs about perceived benefits, including the personal motivation to achieve euphoria, emotional intimacy, increased confidence and decreased inhibitions (42, 44, 47–49). These motives of recreational drug use align with the long-standing four factor model of alcohol motives (59, 60) and five-factor measure for cannabis (61) where people consume these substances to attain the perceived outcomes of intoxication driven by positive or negative internal/external reinforcements.

Mixed results were shared amongst studies and gender differences. Parks and Kennedy (44) and Boys et al. (48), discussed no difference in age of first-time use and number of recreational drugs used across genders whereas other literature acknowledge that men are more likely than women to use all types of illicit drugs (62). The motivational characteristics and desired intoxication effects from use, however, did differ amongst genders. It was reported that motivations to participate in recreational drug use amongst the LGBTQI+ population was more prevalent (50). Boys et al. (48) acknowledged females were more likely to use amphetamines. This finding was supported by two studies where female participants discussed increased their energy levels was a perceived benefit following amphetamine use (63, 64). Furthermore, Fendrich et al.’s (50) findings noting that females suffer more from perceived negative consequences following recreational drug use was supported by Verheyden et al. (65), seeing this prevalent amongst MDMA use.

It is evident that emerging adults are motivated to use illicit drugs in a recreational capacity to improve and enhance personal experiences, most commonly in group settings. Prevalence, and choice of drugs was determined by accessibility and location. The most frequently consumed recreational drugs in the studies included MDMA, LSD, cocaine, ketamine, amphetamines, psilocybin and GHB. The contextual motivations to consume distinct categories of recreational drugs was determined by peers, specific group settings and the intoxication effects participants desired to achieve. Several studies described the specific group settings of recreational drug use. This includes festivals, pubs, clubs, parties and the home environment. Raves and electronic dance music events were identified as an environment for greater consumption of recreational drugs than others who preferred other styles of music (47, 66, 67). This finding is supported by Lim et al. (20), suggesting that different music subcultures were associated with the use of recreational drugs and these settings are underpinned by specific cultural norms as reported by Duff et al. (41). Although types and frequency of drugs used was linked with the different music preferences (20), little is known about the motivational characteristics of use in these social settings. Having a greater understanding of these motivations will enable the implementation of harm reduction interventions appropriate to the environment and the associated risks.

Peer interactions, parties and other social environments contributed to motivational recreational drug use amongst participants. Motivational factors that reinforce emerging adult recreational drug use correlate with the desire to break away from the mundane by seeking pleasure and using drugs for the opportunity to create novel experiences (47, 68). Beliefs about the positive and negative impacts of drug use, together with the motivation to achieve emotional satisfaction influence the motivational characteristics of emerging adults participating in recreational drug use (42).

The availability and accessibility of pill testing was a harm reduction strategy recognized by participants in the studies, but with acknowledgment that it is not always available or beneficial due to the cost and limitations of testing activities (42, 43). However, there was no link to how pill testing has impacted or influenced motivations to use drugs. Current research acknowledges that an abstinence approach is problematic to health policies. Previous harm reduction campaigns have been successful in reducing dangerous levels of alcohol consumption in communities in Germany, Belgium, Czech Republic (69) Sweden (28, 69) and the United States (70). Although these have been successful, it has been identified that these strategies adapted for illicit drug prevention and or reduction campaigns have had minimal impact on decreasing the prevalence illicit drug use.

Similar to alcohol use (59), recreational drug use has been recognized in this review and in current research as a normative behavior amongst emerging adults when attending social settings including clubs and festivals (28, 41, 42, 49, 71). These research findings further support the established concept that a range of factors influence the motivations and behaviors of emerging adults including peer influence, sensation seeking, social norms, social bonding and the media (71–76).

### Recommendations and implications

Several recommendations and implications have emerged from this study. Firstly, conduct further research to investigate how motivational characteristics influence recreational drug use among emerging adults across an array of social settings, with a focus on addressing gaps in knowledge and informing harm reduction strategies and health education. Specifically, explore the role of gender, sexual orientation, and social relationships in relation to recreational drug use.

Secondly, develop a greater understanding of the motivational characteristics of emerging adults engaging in recreational drug use and the societal influences they face. The implication arising is that knowledge gained can inform harm reduction practitioners in developing more culturally and contextually relevant approaches to support safer and informed drug use, thereby reducing adverse harmful outcomes. From this review, it can be acknowledged that the area of motivational characteristics of recreational drug use requires further investigation and research.

Thirdly, a harm reduction and health promotion implication is apparent through engaging with target populations using informed peer-to-peer programs and forums, both formal and informal, as effective strategies for emerging adults to access trustworthy drug information. Such programs should be integrated into future harm reduction practices and research efforts to support users in the informed decision-making process.

Lastly, policy implications arise from our synthesis of studies that confirm that on site pill testing facilities are effective and trustworthy mechanisms for supporting emerging young people with the quality information they require to make informed drug use or non-use decisions within a harm reduction framework. Legislators should consider ways to ensure that large festivals include requirements to accommodate pill testing facilities in the future.

### Limitations

This review has some limitations that need to be acknowledged. Although this study consisted of a comprehensive search strategy, the use of 20-year time frame for the identification of relevant studies may have resulted in using dated literature. According to Whittemore and Knafl (33), a limitation that is also to be acknowledged to a search strategy is the inefficiency of computerized databases, yielding only 50% of eligible studies. This may result in articles not appearing in the search strategy and review because they may have described phenomena using alternative words. Despite the limitation, a strength is that common themes were addressed across multiple studies and in various geographical locales. The themes were consistent and were evident beyond an individual study. Of the 11 studies identified, only three were conducted in the past 10 years, thus demonstrating the need for updated research in this area. This review identified that the literature that is available on this topic is not sufficiently recent to be translated in the contemporary context. Where recent studies exist, they are preliminary and as a result are methodologically weak and contain small sample sizes limiting the generalizability of findings.

## Conclusion

Recreational drug use by emerging adults is strongly driven by the desire to achieve the positive intoxication effects with certain substances aimed at improving and enhancing their personal experiences. Peer influence, social environments and witnessing friends use recreational drugs and observing them enjoy themselves while intoxicated have been identified as playing a crucial role in sourcing drugs, increased curiosity and in the initiation of recreational drug use among emerging adults. Festivals, raves and nightlife settings provide an opportunity to implement health promotion activities that enable a set of interventions to reach large numbers of suitable target individuals within a short period of time. To implement the appropriate actions and information that promotes health messaging and harm minimization interventions for emerging adults, it is important to understand the underlying motivations for use. Study findings synthesized in the review recognized that motivations for drug use stem from the perceived benefits of consumption and personal motives including intoxication, and euphoria. Recommendations of studies from this review acknowledge the need for further research and replication to contribute to contemporary and reliable evidence in this field of study.

## Author contributions

The research question and overall research design and analysis was collaboratively generated, defined and co-drafted by AL, RW, JW, and AH. AH, RW, and JW assisted with design and conceptualization of the study, analysis of data, co-drafted the manuscript and project supervision. AL designed and conceptualized the study, contributed to the literature search, data collection and analysis, writing of the manuscript and critically revised the manuscript. All authors contributed to the article and approved the submitted version.

## References

[1] Australian Government Department of Health and Ageing (AGDHA). Patterns of Use and Harms Associated with Specific Populations of Methamphetamine Users in Australia - Exploratory Research. Canberra: Australian Government Department of Health and Ageing (2009).

[2] European Monitoring Centre of Drugs and Addiction (EMCDDA). European Drug Report Trends and Developments. European Monitoring Centre of Drugs and Addiction. Luxembourg: Office of the European Union (2022).

[3] Arnett J. The developmental context of substance use in emerging adulthood. J Drug Issues. (2005) 35:235–54. doi: 10.1177/002204260503500202

[4] United Nations Office on Drugs and Crime (UNDOC). World Drug Report 2023. Vienna, Austria: United Nations publication (2023). Available at: https://www.unodc.org/unodc/en/data-and-analysis/world-drug-report-2023.html

[5] Australian Institute of Health and Welfare (AIHW). National Drug Strategy Household Survey 2019. Drug Statistics Series No. 32. PHE 270. Canberra, Australia AIHW. (2020). Available at: https://www.aihw.gov.au/getmedia/77dbea6e-f071-495c-b71e-3a632237269d/aihw-phe-270.pdf.aspx?inline=true

[6] Lund A Turris SA. Mass-gathering medicine: risks and patient presentations at a 2-day electronic dance music event. Prehosp Disaster Med. (2015) 30:272–378. doi: 10.1017/S1049023X15004598 25868489

[7] Van Dyck E Ponnet K Van Havere T Hauspie B Dirkx N Schrooten J. Substance use and attendance motives of electronic dance music (EDM) event attendees: a survey study. Int J Environ Res Public Health. (2023) 20:1821. doi: 10.3390/ijerph20031821 36767188 PMC9914168

[8] Weir E. Raves: a review of the culture, the drugs and the prevention of harm. Can Med Assoc J. (2000) 162:1843–8. 10906922 PMC1231377

[9] Hutton A Ranse J Verdonk N Ullah S Arbon P. Understanding the characteristics of patient presentations of young people at outdoor music festivals. Prehosp Disaster Med. (2014) 29:1–7. doi: 10.1017/S1049023X14000156 24555927

[10] Crompton JL. Motivations for pleasure vacation. Ann Tour Res. (1979) 6:408–24. doi: 10.1016/0160-7383(79)90004-5

[11] Cherry K. A. What Is Motivation? Verywell Mind; (2022). Available at: https://www.verywellmind.com/what-is-motivation-2795378. (Accessed May 12, 2022)

[12] Cook DA Artino AR Jr. Motivation to learn: an overview of contemporary theories. Med Educ. (2016) 50:997–1014. doi: 10.1111/medu.13074 27628718 PMC5113774

[13] Nevid JS. Motivation and emotion In: Psychology: Concepts and Applications. 4th ed. Belmont, California, United States of America: Wadsworth Cengage Learning (2013). 286–325.

[14] Hemed E Karsh N Mark-Tavger I Eitam B. Motivation(s) from control: response-effect contingency and confirmation of sensorimotor predictions reinforce different levels of selection. Exp Brain Res. (2022) 240:1471–97. doi: 10.1007/s00221-022-06345-3 35316354

[15] Sjoerds Z Luigjes J van den Brink W Denys D Yücel M. The role of habits and motivation in human drug addiction: a reflection. Front Psych. (2014) 5:1–5. doi: 10.3389/fpsyt.2014.00008 PMC3905212 24523702

[16] Newcomb MD Chou C-P Bentler PM Huba GJ. Cognitive motivations for drug use among adolescents: longitudinal tests of gender differences and predictors of change in drug use. J Couns Psychol. (1988) 35:426–38. doi: 10.1037/0022-0167.35.4.426

[17] Oetting ER Beauvais F. Peer cluster theory, socialization characteristics, and adolescent drug use: a path analysis. J Couns Psychol. (1987) 34:205–13. doi: 10.1037/0022-0167.34.2.205

[18] Segal B Huba GJ Singer J. Reasons for drug and alcohol use by college students. Int J Addict. (1980) 15:489–98. doi: 10.3109/10826088009040032 6967862

[19] Starobin B Jablonski S Andrele AM Powers JB. Recreational drugs: effects on the heart and cardiovascular system in Encyclopedia of Cardiovascular Research and Medicine. 1st Edn. Eds. Vasan R. S. Sawyer D. B. (Elsevier). (2018) 240–248.

[20] Lim M Hellard M Hocking J Aitken C. A cross-sectional survey of young people attending a music festival: associations between drug use and musical preference. Drug Alcohol Rev. (2008) 27:439–41. doi: 10.1080/09595230802089719 18584396

[21] Peacock A Karlsson A Uporova J Gibbs D Swanton R Kelly G. Australian Drug Trends 2019: Key Findings from the National Ecstasy and Related Drugs Reporting System (EDRS) Interviews. UNSW Sydney, Australia: National Drug & Alcohol Research Centre (2019).

[22] Scott I Scott R. Pill testing at music festivals: is it evidence-based harm reduction? Int Med J. (2020) 50:395–402. doi: 10.1111/imj.14742 31908122

[23] Glover T. D. Raves/Raving. Retrieved from Encyclopedia of Recreation and Leisure in America; (2019). Available at: https://www.encyclopedia.com/humanities/encyclopedias-almanacs-transcripts-and-maps/ravesraving (Accessed April 5, 2023).

[24] King G. EDM/Rave Culture. Retrieved from Subcultures and Sociology - Grinnell College; (2017). Available at: https://haenfler.sites.grinnell.edu/subcultures-and-scenes/edmrave-culture/ (Accessed April 5, 2023).

[25] Alcohol and Drug Foundation. What Is Harm Reduction and Why Is It Important at Music Venues and Events? In Staying Safe at Events; (2021). Available at: https://cdn.adf.org.au/media/documents/ADF_StayingSafeAtEvents.pdf (Accessed April 5, 2023).

[26] Hutton A. The role of harm minimisation to prevent alcohol and drug misuse at outdoor music festivals In: Mair J, editor. The Routledge Handbook of Festivals: New York, United States of America: Routledge Handbooks (2018). 92–101.

[27] Hutton A Munn MB White S Kara P Ranse J. Does the presence of on-site medical Services at Outdoor Music Festivals Affect Attendees’ planned alcohol and recreational drug use? Prehosp Disaster Med. (2021) 36:403–7. doi: 10.1017/S1049023X21000613 34187607

[28] Strandberg A Elgán T Feltmann K Lindström N Gripenberg J. Illicit drugs in the nightlife setting: changes in employee perceptions and drug use over a fifteen-year period. Subst Use Misuse. (2020) 55:2116–28. doi: 10.1080/10826084.2020.1793365 32811266

[29] Day N Criss J Griffiths B Gujral S John-Leader F Johnston J. Music festival attendees' illicit drug use, knowledge and practices regarding drug content and purity: a cross-sectional survey. Harm Reduct J. (2018) 15:1–8. doi: 10.1186/s12954-017-0205-7 29304871 PMC5756357

[30] Hughes C Barratt M Ferris J Winstock A. Australian Music Festival Attendees: A National Overview of Demographics, Drug Use Patterns, Policing Experiences and Help-Seeking Behaviour. Sydney, Australia: DPMP (2019).

[31] Miller P Curtis A Jenkinson R Droste N Bowe S Pennay A. Drug use in Australian nightlife settings: estimation of prevalence and validity of self-report. Addiction. (2015) 110:1803–10. doi: 10.1111/add.13060 26189494

[32] Van Havere T Vanderplasschen W Lammertyn J Brokekaert E Bellis M. Drug use and nightlife: more than just dance music. Subst Abuse Treat Prev Policy. (2011) 6:11. doi: 10.1186/1747-597X-6-18 21794101 PMC3160361

[33] Whittemore R Knafl K. The integrative review: updated methodology. J Adv Nurs. (2005) 52:546–53. doi: 10.1111/j.1365-2648.2005.03621.x 16268861

[34] Russell C. An overview of the integrative research review. Prog Transplant. (2005) 15:8–13. doi: 10.1177/152692480501500102 15839365

[35] Page MJ McKenzie JE Bossuyt PM Boutron I Hoffmann TC Mulrow CD. The PRISMA 2020 statement: an updated guideline for reporting systematic reviews. BMJ. (2021) 372:n71. doi: 10.1136/bmj.n71 33782057 PMC8005924

[36] Arnett J. Emerging Adulthood: The Winding Road from the Late Teens through the Twenties. 2nd ed. New York, New York, United States of America: Oxford University Press (2014).

[37] Konstam V. Emerging and young adults: an introduction In: Konstam V, editor. Emerging and Young Adulthood. 2nd ed. Switzerland: Springer (2015)

[38] Lally M Valentine-French S. Emerging and early adulthood In: Lifespan Development A, editor. Psychological Perspective. 2nd ed. California, United States of America: Creative Commons (2019). 246–95.

[39] Jessor R. Risk behavior in adolescence: a psychosocial framework for understanding and action. J Adolesc Health. (1991) 12:597–605. doi: 10.1016/1054-139X(91)90007-K 1799569

[40] Leather NC. Risk-taking behaviour in adolescence: a literature review. J Child Health Care. (2008) 13:295–304. doi: 10.1177/1367493509337443 19713410

[41] Duff C. Johnston J. Moore D. Netzach G. Dropping, Connecting, Playing and Partying: Exploring the Social and Cultural Contexts of Ecstasy and Related Drug Use in Victoria. Victoria. Melbourne, Victoria: Victorian Department of Human Services. (2007) 87–96. Available at: https://www.vgls.vic.gov.au/client/en_AU/search/asset/1161131/0

[42] Fox J Smith A Yale A Chow C Alaswad E Cushing T. Drugs of abuse and novel psychoactive substances at outdoor music festivals in Colorado. Subst Use Misuse. (2018) 53:1203–11. doi: 10.1080/10826084.2017.1400067 29148866 PMC5935531

[43] Levy K O'Grady K Wish E Arria A. An in-depth qualitative examination of the ecstasy experience: results of a focus group with ecstasy-using college students. Subst Use Misuse. (2005) 40:1427–41. doi: 10.1081/JA-200066810 16048826 PMC2948966

[44] Parks K Kennedy C. Club drugs: reasons for and consequences of use. J Psychoactive Drugs. (2004) 36:295–302. doi: 10.1080/02791072.2004.10400030 15559677

[45] Peters G Kok G Schaalma H. Careers in ecstasy use: do ecstasy users cease of their own accord? Implications for intervention development. BMC Public Health. (2008) 8:376. doi: 10.1186/1471-2458-8-376 18957117 PMC2583996

[46] Ter Bogt T Engels R. "partying" hard: party style, motives for and effects of MDMA use at rave parties. Subst Use Misuse. (2005) 40:1479–502. doi: 10.1081/JA-200066822 16048829

[47] Biolcati R Mancini G. Club drugs and rave parties: a pilot study on synthetic drug consumption styles in a sample of young Italian ravers. Open Public Health J. (2018) 11:474–84. doi: 10.2174/1874944501811010474

[48] Boys A Marsden J Strang J. Understanding reasons for drug use amongst young people: a functional perspective. Health Educ Res. (2001) 16:457–69. doi: 10.1093/her/16.4.457 11525392

[49] Duff C. Party drugs and party people: examining the ‘normalization’ of recreational drug use in Melbourne, Australia. Int J Drug Policy. (2005) 16:161–70. doi: 10.1016/j.drugpo.2005.02.001

[50] Fendrich M Wislar JS Johnson TP Hubbell A. A contextual profile of club drug use among adults in Chicago. Addiction. (2003) 98:1693–703. doi: 10.1111/j.1360-0443.2003.00577.x, PMID: 14651501

[51] Smirnov A Najman J Hayatbakhsh R Plotnikova M Wells H Legosz M. Young adults' trajectories of ecstasy use: a population based study. Addict Behav. (2013) 38:2667–74. doi: 10.1016/j.addbeh.2013.06.018 23899430

[52] Critical Appraisal Skills Program (CASP). CASP Checklist; (2018). Available at: https://casp-uk.net/casp-tools-checklists/ (Accessed August 3, 2022)

[53] Parahoo K. Research designs In: Parahoo K, editor. Nursing Research. 2nd ed. New York: Palgrave Macmillan (2006). 190.

[54] World Health Organization (WHO). Multiple Drug Use. Lexicon of Alcohol and Drug Terms. Geneva: WHO (1994). 46 p.

[55] Miller L. American Addiction Centers: Stimulant Drug Abuse. San Diego: American Addiction Centers (2022).

[56] Sheidow AJ McCart M Zajac K Davis M. Prevalence and impact of substance use among emerging adults with serious mental health conditions. Psychiatr Rehabil J. (2012) 35:235–43. doi: 10.2975/35.3.2012.235.243 22246122 PMC3767039

[57] Schulenberg JE Johnston LD O’Malley PM Bachman JG Miech RA Patrick ME. Monitoring the Future National Survey Results on Drug Use, 1975–2018: Vol. II, College Students and Adults Ages 19–60. Michigan, United States of America: Institute for Social Research, The University of Michigan. (2019). Available at: 10.3998/2027.42/150623

[58] Spoth R Redmond C Shin C Trudeau L Greenberg MT Feinberg ME. Applying the PROSPER prevention delivery system with middle schools: emerging adulthood effects on substance misuse and conduct problem behaviors through 14 years past baseline. Child Dev. (2022) 93:925–40. doi: 10.1111/cdev.13746 35289921 PMC9543769

[59] Cooper ML. Motivations for alcohol use among adolescents: Development and validation of a four-factor model. Psychol Assess. (1994) 6:117–28. doi: 10.1037/1040-3590.6.2.117

[60] Cox WM Klinger E. A motivational model of alcohol use. J Abnorm Psychol. (1988) 97:168–80. doi: 10.1037/0021-843X.97.2.168 3290306

[61] Simons J Correia CJ Carey KB Borsari BE. Validating a five-factor marijuana motives measure: relations with use, problems, and alcohol motives. J Couns Psychol. (1998) 45:265–73. doi: 10.1037/0022-0167.45.3.265

[62] Center for Behavioral Health Statistics and Quality. Results from the 2016 National Survey on Drug Use and Health: Detailed Tables. Rockville, MD: Substance Abuse and Mental Health Services (2017).

[63] Brecht ML O'Brien A von Mayrhauser C Anglin MD. Methamphetamine use behaviors and gender differences. Addict Behav. (2004) 29:89–106. doi: 10.1016/s0306-4603(03)00082-0 14667423

[64] Cretzmeyer M Sarrazin MV Huber DL Block RI Hall JA. Treatment of methamphetamine abuse: research findings and clinical directions. J Subst Abus Treat. (2003) 24:267–77. doi: 10.1016/s0740-5472(03)00028-x 12810148

[65] Verheyden SL Hadfield J Calin T Curran HV. Sub-acute effects of MDMA (+/−3,4-methylenedioxymethamphetamine, "ecstasy") on mood: evidence of gender differences. Psychopharmacology. (2002) 161:23–31. doi: 10.1007/s00213-001-0995-9 11967627

[66] De Almeida SP Silva MT. Characteristics of ecstasy users in Sãio Paulo, Brazil. Subst Use Misuse. (2005) 40:395–404. doi: 10.1081/ja-200052290 15776985

[67] Forsyth AJM Barnard M McKeganey NP. Musical preference as an indicator of adolescent drug use. Addiction. (1997) 92:1317–25. doi: 10.1111/j.1360-0443.1997.tb02850.x 9489048

[68] Kennett J Matthews S Snoek A. Pleasure and addiction. Front Psych. (2013) 4:117. doi: 10.3389/fpsyt.2013.00117 PMC3782756 24093020

[69] Arnaud N Baldus C Elgan TH De Paepe N Tonnesen H Csemy L. Effectiveness of a web-based screening and fully automated brief motivational intervention for adolescent substance use: a randomized controlled trial. J Med Internet Res. (2016) 18:e103. doi: 10.2196/jmir.4643 27220276 PMC4897296

[70] White HR Jiao Y Ray AE Huh D Atkins DC Larimer ME. Are there secondary effects on marijuana use from brief alcohol interventions for college students? J Stud Alcohol Drugs. (2015) 76:367–77. doi: 10.15288/jsad.2015.76.367 25978822 PMC4440295

[71] Jaensch J Whitehead D Prichard I Hutton A. Exploring young peoples’ use of alcohol at outdoor music festivals in Australia. J Appl Youth Stud. (2018) 2:32–42. doi: 10.3316/informit.431681436145440

[72] Bradley G Wildman K. Psychosocial predictors of emerging adults’ risk and reckless behaviors. J Youth Adolesc. (2002) 31:253–65. doi: 10.1023/A:1015441300026

[73] Glassman T Braun RE Dodd V Miller JM Miller EM. Using the theory of planned behaviour to explain the drinking motivations of social, high-risk, and extreme drinkers on game day. J Community Health. (2010) 35:172–81. doi: 10.1007/s10900-009-9205-1 20013062

[74] Hutton A Roderick A Munt RML Kako M Arbon P. Celebrating the end of school life: a pilot study. Prehosp Disaster Med. (2012) 27:13–7. doi: 10.1017/S1049023X11006765 22591925

[75] McCreanor T Moewaka Barnes H Kaiwai H Borell S Gregory A. Creating intoxigenic environments: marketing alcohol to young people in Aotearoa New Zealand. Soc Sci Med. (2008) 67:938–46. doi: 10.1016/j.socscimed.2008.05.027 18619720

[76] Teese R Bradley G. Predicting recklessness in emerging adults: a test of a psychosocial model. J Soc Psychol. (2008) 148:105–26. doi: 10.3200/SOCP.148.1.105-128 18476486

