# Supplementary material for: Motivational characteristics of recreational drug use among emerging adults in social settings: an integrative literature review
Source: Front Public Health. 2023 Oct 31;11:1235387. doi: 10.3389/fpubh.2023.1235387 (PMC10644826; doi:10.3389/fpubh.2023.1235387)
Supplement: Supplementary file 1 [file Table_1.docx]

**Supplementary Table 1:** Summary of Studies

| Author/ Year/  Title | Study  Setting | Study  Type | METHODS | Number of Participants | | FINDINGS | LIMITATIONS | RECOMMENDATIONS |
| --- | --- | --- | --- | --- | --- | --- | --- | --- |
|  |  |  |  | M | F |  |  |  |
| (1)  (Biolcati & Mancini, 2018)  (47)  Club Drugs and Rave Parties- A Pilot Study on Synthetic Drug Consumption Styles in a Sample of Young Italian Ravers | Bologna, Italy  5 nightclubs | Quantitative  Ethnographic method | Targeted sampling  Ad hoc questionnaire | 23 | 14 | 37 users  Aged 22–33  (M = 26.57, SD = 3.02)   - Recreational drug use: - Consumed MDMA, Speed, LSD - highly declared rates - Participants chose substances belonging to the same drug group for specific and contextual motives. - Drug use settings – group settings - raves and parties (28.9%); discos (20.7%); private parties (19.0%); around friends (14.9%); at home (13.2%); at work (3.3%)   Concurrent drug use:   - High frequency of concurrent drug use by participants - Desirable motivation reinforces   Feel fit and stronger, state of trance, in tune with others, not thinking about problems, doing things I would otherwise not do, transgressing  Peer and social influence:   - Increased curiosity & socialization | - Pilot Study in Italy – limited sample size– not generalizable - Ad hoc self-report only allows first exploratory survey and lacks information related to dependence, health status, personality variables which could better clarify vulnerability of sample. - Correlational investigations and comparison between groups only allows co-occurrences analysis – insufficient in investigating casual relationships | - Future surveys should contribute to this field by studying target groups such as ravers - New methodological frame – structured assessment tools |
| (2)  (Boys et al., 2001)  (48)  Understanding reasons for drug use amongst young people: a functional perspective | United Kingdom | Quantitative  Functional Perspective | Snowball-sampling approach  Structured Interviewer questionnaire audiotaped | 205 | 159 | 364 poly-drug users  Aged 16–22 years  (Mean age: 19.3 years)  Ethnicity:  White-European 69.8%  Black 12.6%  Asian 10.1%  Recreational drug use:   - All 6 drugs had been used to fulfil all functions measured, despite differences in pharmacological effects   Desirable motivation reinforces:   - Most popular functions for use: relax (96.7%), become intoxicated (96.4%), keep awake at night while socializing (95.9%), enhance an activity (88.5%), alleviate depressed mood (86.8%). | - Published 2001 - Snowball-sample methodology – not random sample research | - Further research required to determine whether observations may be generalized to other populations & drug types or if additional function items need to be developed. - Examine if functions can be categorized into primary and subsidiary reasons for use & how these relate to changes in patterns of use and dependence - Recognition of functions fulfilled by substance use could help inform education & prevention strategies to make approaches more relevant & acceptable to target audiences |
| (3)  (Duff, 2005)  (49)  Party drugs and party people: examining the ‘normalization’ of recreational drug use in Melbourne, Australia | Melbourne,  Australia  Bars & Nightclubs | Quantitative  Cross-sectional | Intercept surveys | 209 | 170 | 379 bar and nightclub patrons  Aged 18-44  (mean: 22.9 years, mode: 20)  Recreational drug use:   - Prevalence of drugs attributed to research setting - Use of drugs found to be widespread - 56% ‘lifetime use’, 35.2% recent (last month) use. Typically: ecstasy, cannabis and/or amphetamines. - Poly-drug use common with majority of participants. - Young people organize/stagger drug use across evening – starting with alcohol before using psychostimulants & finishing with alcohol & cannabis   Undesirable motivation reinforces   - Loss of memory, depression, loss of coordination, relationship difficulties   Normalization of use:   - Drug use is becoming increasingly normalized within youth populations in Australia. | - Published 2005 - Research narrowly focused - Small sample size- limiting definitive conclusions | - To understand young people’s drug use – consider broad cultural studies of drug use. - Consider change in young people’s leisure time, including growing interest in clubbing, the ‘night-time economy’, & impacts on the cultures and contexts of young people’s drug use. - Study of drug use requires new approaches - that go beyond conventional epidemiological accounts of incidence & prevalence of young people’s drug use - Future approaches should focus more on attempting to reduce the harms associated with young people’s drug use than on attempting to eliminate use in total. - Experiment with research methodologies in development of effective evidence base to guide future policy directions. - New types of information and peer education strategies will be required in specific ‘drug use settings’ such as bars, nightclubs, the workplace, schools & universities. |
| (4)  (Duff et al., 2007) (41)  Dropping, Connecting, Playing and Partying: Exploring the social and cultural contexts of ecstasy and related drug use in Victoria | Victoria, Australia    Observational data: | Qualitative  Theoretical sampling  Iterative & purposive sampling  grounded theory, participatory action research and ethnography | Indigenous fieldworkers, observational studies, research interviews, in-depth case studies | 30 | 22 | 52 in-depth interviews completed with current and active ecstasy and related drugs users.  Age 18-36 years (average 23)  Recreational drug use:   - Drug use reported differently according to circumstances & cultural contexts – variations between rural, regional & metropolitan settings & between different youth subcultures & scenes. - Concurrent drug use   Consumer drug knowledge:   - Strategies employ to reduce/manage harms - Emerging adults aware of risks and dangers associated to drug use - Participants identified need for all drug prevention programs & strategies to be factual, balanced and credible. - Levels of harm reduction knowledge appeared to variate over time – increases with continued use & shared knowledge from peers   Desirable motivation reinforces   - Enhanced experiences and intimacy, opportunities for connections, euphoria, curiosity, peer enjoyment   Undesirable motivation reinforces:   - The comedown, depression, stress on relationships   Normalization of use/drug use settings:   - Recreational drug use – growing in normalization in Victoria – within club, bar & rave scenes   Peer and social influence:   - The influence of peer networks is central to the experience of use in Melbourne – providing information, facilitating access to sources & shaping ways consumed | Published 2007  No limitations documented | - Further research required to confirm how widespread attitudes might be among the broader youth population - study findings are consistent with other Australian studies - Ongoing research needed – to improve the quality of existing policy interventions. - Vital need to better understand users’ perceptions, values and needs. - ideally designed to complement existing monitoring projects such as: Party Drugs Initiative, Premier’s Drug Prevention Council Victorian Youth Alcohol and Drug Survey - Cultural contextualization of risk and harm associated to drug use requires further research - Prevention interventions – sensitive to relevant cultural and contextual differences – populations and geographical regions |
| (5)  (Fendrich et al., 2003) (50)  A contextual profile of club drug use among adults in Chicago | Chicago,  United States of America | Quantitative  Cross-sectional  Participants drawn randomly from selected households using multi-stage area probability design | Audio Computer-Assisted Self interview and optional drug screening sample | 242 | 385 | 627 participants  Aged 18-40  38.9% aged 18 - 25  26.2% aged 26 - 30  34.9% aged 31-40 Ethnicity:  African Americans 40.6%  White 32%,  Hispanic 18.2%  Other 9.2%  Recreational drug use   - Overall club drug prevalence was nearly twice as high as MDMA alone - Use associated with gender, race & sexual orientation   Drug use settings/concurrent drug use:   - Club drug users were more likely to use drugs concurrently & reported treatment for use (25 participants)   Desirable motivation reinforces   - Get high and enjoy themselves, spirit of the party, enjoy sex more, stay up longer | - Published 2003 - Low response rate - High SES respondents likely to be under-represented - Possible sample bias could affect conclusions about risk factors - Findings may be consequence of misinterpretation of questions - Analysis compared club drug users to all study participants - Nonusers treated as homogeneous group and subgroups not analyzed according to use or preference of specific-non club substances - Potential bias from self-reporting | - Continued research required: determine whether findings are generalizable to other populations and settings including: continued biological testing for club drugs in epidemiological surveys - Further research: gender differences, impact of sexual orientation in relation to club drug risks, effects of personality characteristics (sensation seeking) on club drug behaviour - Future studies (club drug use risk) – consider using well-established measures of personality. - Further investigation regarding club drug use among gay and bisexual women - little available information - Future research to identify consumption patterns & risk factors that can be successfully targeted - Raves may provide unique opportunity for communicating harm reduction messages– predisposed towards risk taking behaviors |
| (6)  (Fox, et al., 2018) (42)  Drugs of Abuse and Novel Psychoactive Substances at Outdoor Music Festivals in Colorado | Colorado,  United States of America  Sonic Bloom & Arise Music Festivals | Qualitative  Functional Perspective | Semi-structured qualitative interview  Convenience sampling  Stationed in medical tents | 106 | 65 | 171  Aged 18+ nil max age recorded  (mean 25.5 years) (IQR: 21-29)  Ethnicity:  Caucasian 89.2%  Hispanic 7.8%  Black 2.9%  American Indian/Alaska Native 2.9%  Asian 1 (1%)  Recreational drug use   - MDMA, LSD, cocaine, marijuana – most popular drugs used   Consumer drug knowledge:   - Most users were experienced – perceived minimal risks associated to drug use   Desirable motivation reinforces:   - Primary motivations for use: viewed empathogenic, entactogenic and entheogenic effects of drugs   Undesirable motivation reinforces   - Disorientation, disconnection, temperature dysregulation, jaw tension, negative or depressing thoughts & hangover   Normalization of use:   - Almost all reported normalization of drugs at music festivals | - Cross sectional – conducted in Colorado - Medium-sized sample – limited thorough description of drug patterns and behaviors - Limited capture from festival setting (music festival type) & participant willingness to discuss drug use - Self-presented – possible bias towards negative perceptions - Voluntary participant design – selection bias - Self-reported – potential bias due to limited memory & recall - Prevalence of drug may vary regionally – could affect frequency of drugs reported | - Educational interventions and revised testing strategies may promote safer drug use and reduce adverse outcomes |
| (7)  (Levy et al., 2005)  (43)  An In-Depth Qualitative Examination of the Ecstasy Experience: Results of a Focus Group with Ecstasy-Using College Students | Maryland,  United States of America | Qualitative  Targeted sampling | Focus groups  Short survey and discussion  Respondent driven sampling | 13 | 17 | 30 participants  Aged 18-23 (mean19.5)  Ethnicity:  White 90%  Asian/ Pacific 7%  Black 3%  Recreational drug use: MDMA  Concurrent drug use   - All participants were polysubstance users, consuming other substances simultaneously and concurrently with ecstasy.   Consumer drug knowledge   - Most participants had a basic understanding of the contents of ecstasy pills, and effects from consumption. - Majority: unaware of specific issues caused by used and discounted the potential harms. - Divided response regarding whether ecstasy increased the likelihood of engaging in risky behaviors (sexual) - Positive and negative effects (physical and psychological) that they attributed to their use of ecstasy.   Desirable motivation reinforces   - Positive effects on: mood, social pressure, curiosity, availability, boredom, desire for an altered state of mind, desire to escape, self-medication, desire to have fun, and the ease of use of ecstasy in comparison to other drugs as reasons for initiating ecstasy use.   Undesirable motivation reinforces   - Motivation for quitting ecstasy use varied. Reasons included: negative individual experiences, health concerns, and addiction/tolerance.   Peer and social influence   - Peers appear to have significant impact on the initiation and continued use of ecstasy - When sober & surrounded by ecstasy-using peers whom they perceived to be enjoying themselves, participants described feeling the urge to experience similar high | - Published 2005 - Small and self-selected sample - Unknown if results can be generalizable to other populations | - Replication of methodology in diverse sample of schools needed - Future studies of users who do not attend college required to determine how college environment influences ecstasy use and associated consequences. |
| (8)  (Parks & Kennedy, 2004)  (44)  Club Drugs: Reasons for and Consequences of Use | New York,  United States of America | Qualitative  Randomly selected sampling | preliminary descriptive study  Respondent driven sampling  Face-to-face interviews Audiotaped Open ended questions | 27 | 23 | 50 participants  Aged 18 -30  Average 21.3 years (SD = 3.1).  Ethnicity:  European-American 84%  African American 8%  Other ethnic groups. 8%  Recreational drug use   - Ecstasy was the most frequently used club drug followed by ketamine, LSD and methamphetamine. - All participants reported using club drugs to "experiment"   Drug use settings   - Club drugs were frequently used at raves, in bars or clubs, and at home with friends.   Desirable motivation reinforces   - Most reported using drugs to feel good, enhance social activities & euphoria   Undesirable motivation reinforces   - Negative physical, psychological, and lifestyle consequences were reported for club drug use. - Temperature dysregulation, stomach pains, headaches, memory loss, depression, confusion - Despite negative consequences, participants perceived several positive consequences of regular recreational club drug use. | - Published 2004 - Descriptive nature, limited in size – additional studies needed to confirm and expand findings | - Further exploration of reasons & positive consequences associated to use of each club drugs may provide information on growing trends of use - Future research: explore reasons for club drug use by each drug - Drugs used may provide explanation for the general acceptance of club drug use among young adults. - Future studies of the negative consequences of club drug use needed to employ some form of substance-using comparison group (e.g., cannabis- or alcohol-only users). - Studies with appropriate comparison groups will allow better assessment of negative physical, psychological, & lifestyle consequences associated specifically with use of club drugs. |
| (9)  (Peters et al., 2008)  (45)  Careers in ecstasy use: do ecstasy users cease of their own accord? Implications for intervention development | Maastricht  Netherlands  Dutch Dance Scene | Qualitative  Functional Perspective | Selected random sampling Systematically analyzed -NVivo  5 focus groups discussions  8 individual interviews | 20 | 12 | 32 participants  Ages mentioned 18-25 – (not specified)  Average 21 years  Recreational drug use: ecstasy  Consumer drug knowledge   - During use, participants applied harm reduction strategies, however, strategies were inconsistent and sometimes incorrect - Most appeared to cease ecstasy use automatically – loss of interest or change in life circumstances.   Desirable motivation reinforces   - Most users started out of curiosity & interaction with ecstasy-using friends - Euphoria, energy, connectedness with peers, decreased social inhibitions, ‘levelled’ with using friends   Peer and social influence   - Peer modelling seems to encourage use - ‘Levelled’ with using friends | - Published 2008 - Study relied on self-reports & self-reported casual links between reasons and behaviour   Focus group discussions & individual interviews provided preliminary indications of differences in way participants discussed their (non) drug use, issue has not been explored thoroughly.   - Qualitative methodology suitable for exploratory research | - Urgent need for more research into the effectiveness of different harm reduction strategies - Requires other studies to see if patterns emerge over time - Implementation of harm reduction strategies may be enabled or enhanced by improved party conditions, (eg providing on site testing facilities, free water distribution, organized chilling breaks, prompts to drink, eat & chill out) - Requires quantitative verification of the results to allow statistical inference & generalization. |
| (10)  (Smirnov et al., 2013) (51)  Young adults' trajectories of Ecstasy use: A population based study | Queensland  Australia | Quantitative study of Mixed-Method Study  Population-based Retrospective/ prospective cohort study | Population screening  Trajectories derived from cluster analysis  Two face-to-face interviews  Two online surveys | N/A | N/A | 297 participants  Aged 19–23 years  Recreational drug use:   - Three Ecstasy trajectories were identified (low, intermediate and high use) - High-use trajectory: 1–2 days of use per week. - Intermediate & high-use trajectory predicted by past Ecstasy consumption (>70 pills) & attendance at electronic/dance music events. - High-use trajectory members - unlikely to have used Ecstasy for more than 3 years & tended to report consistently positive subjective effects at baseline. - Given social context & temporal course of ecstasy use, trajectories may be better understood in terms of instrumental rather than addictive drug use patterns.   Desirable motivation reinforces   - Euphoria, increased confidence, increased libido, being talkative, being very friendly & increased empathy/understanding.   Undesirable motivation reinforces   - Nervousness, panic attacks/anxiety, paranoia, irritability, aggression/hostility, hallucinations/delusions, tension, depression & mood swings   Peer and social influence   - Use largely motivated by social-recreational goals. - Recurrently attended electronic/dance music events were almost 3 times more likely than other participants to be part of the intermediate and high-use trajectories. | - Low screening response rate - Potential bias from non-response - Power limitations – sample size may limit capacity to identify significant associations - Cohort specific study - Gaps in 12- & 30-month data collection - Potentially short data collection timeframe – potential to evaluate possible long-term problematic use - Did not assess drug and alcohol treatment or drug education campaigns as influences of decrease in use - Trajectory groups are statistically derived rather than theory-based. Do not represent a formal classification. | - Future research should evaluate the effects of interventions on the natural history of Ecstasy use. - More research required to understand the extend of long-term neuropsychological harm results from intensive short-term use |
| (11)  (Ter Bogt & Engels, 2005)  (46)  “Partying” Hard: Party Style, Motives for and Effects of MDMA Use at Rave Parties | Amsterdam  Netherlands | Quantitative  Functional Perspective  Observational | Random sampling | 323 | 167 | 490 participants  Aged 14–43 years  (mean 22.3 years, SD = 5.03)  Recreational use: MDMA  Concurrent use:   - MDMA used with: cannabis, psylocibin, speed, cocaine - Quantity of MDMA use -associated with hardcore and trance/mainstream party style,   Desirable motivation reinforces   - Partygoers primarily motivated by energetic & euphoric effects expected from MDMA - Motives of euphoria, sexiness, self-insight, and sociability/flirtatiousness, energy   Undesirable motivation reinforces   - Confusion, being out of control, nausea, suspiciousness, edginess, dizziness, aggression, fear, headaches, fainting - Women report more (acute) negative effects than men especially those motivated to cope with problems by using. - Men’s polydrug use and notably their motivation to conform to friends by using MDMA are associated with negative effects.   Peer and social influence   - Motivated to conform with friends - Increased use when MDMA is used by peers | - Published 2005 - Validity of self-reports and intoxication - Inability to follow-up tests as participants did not want to provide personal details - Possible underrepresentation of group – risk of systematically missing parts of audience - Absence of groups of ‘heavy’ use as too intoxicated to complete questionnaire – no reliable conclusion drawn on quantity & frequency of drug use | - Research in party scenes is rare (raves) - Research should try to adapt to the conditions and not be made impossible by rigidly applied methodological concerns |
